# A High-Voltage-Isolated MEMS Quad–Solenoid Transformer with Specific Insulation Barriers for Miniaturized Galvanically Isolated Power Applications

**DOI:** 10.3390/mi15020228

**Published:** 2024-01-31

**Authors:** Changnan Chen, Pichao Pan, Jiebin Gu, Xinxin Li

**Affiliations:** 1 State Key Laboratory of Transducer Technology, Shanghai Institute of Microsystem and Information Technology, Chinese Academy of Sciences, Shanghai 200050, China; ccn@mail.sim.ac.cn (C.C.); panpichao@mail.sim.ac.cn (P.P.); j.gu@mail.sim.ac.cn (J.G.); 2 University of Chinese Academy of Sciences, Beijing 100049, China

**Keywords:** micro-transformer, galvanic isolation, electromagnetic coupling, MEMS micro-casting technique, monolithic integration, power supply on chip

## Abstract

The paper reports on high voltage (HV)-isolated MEMS quad–solenoid transformers for compact isolated gate drivers and bias power supplies. The component is wafer-level fabricated via a novel MEMS micro-casting technique, where the tightly coupled quad–solenoid chip consists of monolithically integrated 3D inductive coils and an inserted ferrite magnetic core for high-efficiency isolated power transmission through electromagnetic coupling. The proposed HV-isolated transformer demonstrates a high inductance value of 743.2 nH, along with a small DC resistance of only 0.39 Ω in a compact footprint of 6 mm^2^, making it achieve a very high inductance integration density (123.9 nH/mm^2^) and the ratio of *L/R* (1906 nH/Ω). More importantly, with embedded ultra-thick serpentine-shaped (S-shaped) SiO_2_ isolation barriers that completely separate the primary and secondary windings, an over 2 kV breakdown voltage is obtained. In addition, the HV-isolated transformer chips exhibit a superior power transfer efficiency of over 80% and ultra-high dual-phase saturation current of 1.4 A, thereby covering most practical cases in isolated, integrated bias power supplies such as high-efficiency high-voltage-isolated gate driver solutions.

## 1. Introduction

With the newly emerging dense-power, high voltage-level applications such as electric vehicles (EVs) [1], fast-charging uninterruptible power supplies (UPSs) [2], and renewable energy microgrids [3,4] rapidly evolving, the energy density, high-power handling capability, and robustness of the highly miniaturized power converters utilized in these applications have become more critical. Recent advancements in wide-bandgap (WBG) semiconductor material-based devices, including gallium nitride (GaN) high electron-mobility transistors and silicon carbide (SiC) metal–oxide–semiconductor field-effect transistors (MOSFETs) which show superior electric and thermal characteristics [5], have largely helped eliminate the bottleneck of power modules by dramatically maximizing the converter efficiency and power density at the higher switching frequencies and operating voltages [6]. However, in WBG switching device-based power converter applications, electric components in the low-voltage domain are susceptible to voltage surges, ground drift, and leakage current introduced from power MOSFETs in the high-voltage domain, which can potentially cause a breakdown of integrated control circuits [7,8]. Therefore, as the interface between the low-voltage controller and the high-voltage power stage, the integrated micro-transformers that power the gate drivers through embedded galvanic isolation barriers [9] to control the energy flow in WBG switching devices are required to protect against large potential differences, which can be as high as 800 V [10]. Moreover, safety regulations for electronics and human-operated equipment always require galvanic isolations [11], since high-voltage electric contact accidents can trigger electric shock and potentially cause fatal ventricular fibrillation.

Traditionally, optocouplers and enameled wire-wound discrete transformers have been adopted for isolated data transfer [12] and isolated power conversion [13], respectively, in order to provide full isolation. However, these discrete devices, which are bulky and unable to be integrated on-chip, are failing to meet the emerging demands for the miniaturization and integration of dense-power systems. With the rapid development of advanced electronic packaging and MEMS microfabrication technology, on-chip integrated micro-transformers with minimal footprint for compact galvanically isolated gate driver applications are highly desired. Unfortunately, integrating transformers monolithically and achieving high-voltage isolation above 2 kV while maintaining high-power transfer efficiency is still challenging due to the thickness of insulating materials and the structurally constrained coupling coefficient. For example, monolithic air-core transformers with planar interleaved coupling coils, which are simple to micromachine, have been implemented for isolated power transfer in [14,15,16] but are restricted with lower isolation voltage ratings of only 20 V, 200 V, and 380 V, respectively. The limitations are attributed to the incomplete galvanic isolation between the coupling coils and the substrate, as well as the thinness and low dielectric strength of the dielectric layer used as an isolation barrier. In addition, the relatively limited inductance integration density (e.g., 8.0 nH/mm^2^ [17]) that the monolithic air-core transformers can offer restricts the periodic magnetic energy storage, and thus leads to a higher operating frequency of above 100 MHz, thereby degrading the system efficiency through substantial switching and rectification losses. Efforts have been made to increase the inductance value of micro-transformers by wrapping high-permeability magnetic materials outside the racetrack-shaped coupling coils [18,19] or by electroplating thin-film magnetic cores inside solenoidal coils [20,21], both of which multiply the fabrication complexity. Additionally, subsequent high-temperature processes pose inherent degradation in the permeability of these pre-deposited magnetic cores, which are already limited in thickness (e.g., only 3 μm thick in [22] and 6 μm thick in [20]), resulting in inadequate magnetic flux enhancement and, thus, limited inductance increments, and making them unfavorable for further improvements in efficiency and integration.

Therefore, aiming to significantly enhance the galvanic isolation capability and inductance integration density of monolithically integrated micro-transformers in a targeted operating frequency of a few megahertz, in this paper, one kind of novel structure design of three-dimensional quad–solenoid coupling coils with serpentine-shaped (S-shaped) ultra-thick SiO_2_ isolation barriers and unique fabrication processes for high-volage-isolated silicon-integrated micro-transformers are proposed.

## 2. Design and Modeling

### 2.1. Structural Design of the High-Voltage-Isolated Transformer Device

**Figure 1:**
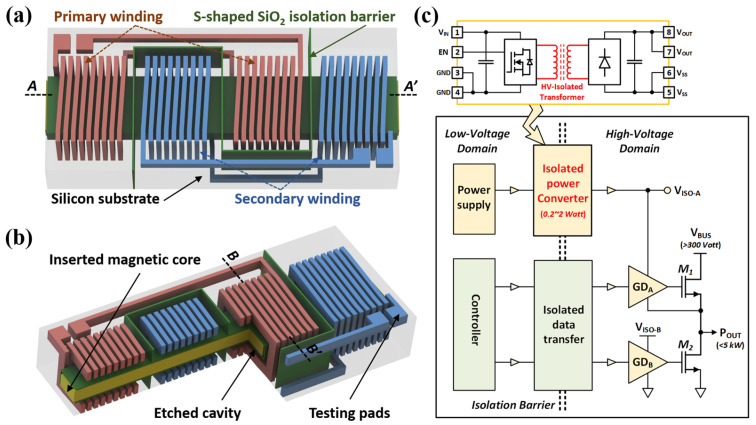
(**a**) Schematic three-dimensional illustrations of the proposed quad–solenoid HV-isolated transformer chip and its (**b**) cross-sectional view, demonstrating the inserted ferrite magnetic core. (**c**) Simplified electrical architecture of fully isolated gate driver solutions with integrated isolated data and power transfer.

The detailed structure design and the isolated power transfer functionalities of the proposed high-voltage-isolated MEMS quad–solenoid transformer for compact galvanically isolated gate driver applications are schematically shown in Figure 1. Two sets of primary and secondary coupling coils are embedded inside the high-resistance silicon substrate in the form of a comb-like interleaved arrangement. Specifically, the primary and secondary windings are composed of series-connected dual solenoids, respectively, so together, they form a tightly coupled quad–solenoid structure to enable high-efficiency isolated power transmission at the targeted operating frequency of around 3 MHz through electromagnetic coupling. In addition, as indicated in Figure 1a, ultra-thick S-shaped SiO_2_ isolation barriers with high dielectric strength completely separate the primary and secondary windings from the high-resistance silicon substrate on which they are embedded, thus ensuring high-voltage isolation.

As for the metallization of three-dimensional solenoidal coupling coils, traditionally, the use of multiple photolithography and micro-electroplating is the only way to form such complex multilayer metal structures with through-silicon vias (TSVs) connecting the upper and lower metal grooves, inevitably introducing high contact resistance between the metal layers. As an alternative to the time-consuming micro-electroplating process, the molten alloy MEMS micro-casting technique [23] was first developed by our group, aiming to enable advanced IC packaging with dense vertical electric interconnections [24]. Herein, this novel Zn-Al alloy micro-casting process is further optimized to facilitate the rapid metallization of a three-dimensional high-density solenoidal structure within the pre-etched silicon-mold wafers. Benefiting from the micro-casted thick-metal structures with high aspect ratios, the silicon-embedded solenoidal windings take advantage of the high coil density and ultra-low DC resistance, which further increases the quality factor (*Q*-factor). The MEMS micromachining process for the formation of silicon-mold wafers and the scheme of the subsequent micro-casting process are detailed in Section 3. Additionally, as shown in Figure 1b, the coupling solenoidal windings in the HV-isolated transformer take full advantage of the effective magnetic flux enhancement introduced by the inserted high-permeability 200 micron-thick ferrite magnetic core to significantly increase the inductance values and largely eliminate leakage inductance. Therefore, a high inductance integration density and coupling coefficient are ensured. In addition, the unclosed magnetic core configuration allows for a high saturation current of the HV-isolated transformer.

### 2.2. Device Modeling

The inductance of the coupling solenoidal coils in the designed HV-isolated transformer chips with an inserted ferrite magnetic core can be analytically quantified from the combination of air core inductance, *L_air_*, and the inductance increment introduced from the magnetic core, Δ*L*, based on the following equations [25]:

(1) $$L_{mag}=L_{air}+\Delta L$$

where the air core inductance, *L_air_*, can be further obtained based on the equation in [26] as

(2) $$L_{air}=\frac{\mu_{0}\mu_{r\_air}N^{2}S}{l}$$

where *μ_r_air_* is the relative permeability of air, *l* represents the axial length of the solenoidal coils, and *S* is the inner cross-sectional area of the solenoidal coils. The total number of turns of the coupling primary or secondary windings in the 1:1 transformer is written as *N*. Since the inductive windings are composed of series-connected dual solenoids, the above equation can be rewritten as

(3) $$L_{air}=\frac{\mu_{0}\mu_{r\_air}N_{1}^{2}S}{l_{1}}+\frac{\mu_{0}\mu_{r\_air}N_{2}^{2}S}{l_{2}}$$

The numbers of turns of individual windings in the series-connected dual solenoids are *N*_1_ and *N*_2_, respectively; and the axial lengths are *l*_1_ and *l*_2_, respectively. In addition, the magnetic core-introduced inductance increment, Δ*L*, can be approximated by [25]

(4) $$\Delta L=\frac{\mu_{0}\mu_{rc}N^{2}w_{mag}t_{mag}}{l_{mag}\left[1+N_{d}\left(\mu_{rc}-1\right)\right]}=\frac{\mu_{0}\mu_{eff}N^{2}w_{mag}t_{mag}}{l_{mag}},\;\mu_{eff}=\frac{\mu_{rc}}{\left[1+N_{d}\left(\mu_{rc}-1\right)\right]}$$

where *μ_rc_* is the relative permeability of the inserted magnetic core; and *w_mag_*, *t_mag_*, and *l_mag_* are the core’s width, thickness, and length, respectively. Since the demagnetizing field inside the finite-sized magnetic core will effectively reduce the core’s relative permeability, the demagnetizing factor *N_d_*, proposed by D.-X. Chen in [27], with its numerical solutions available in [28], is adopted to characterize the effective permeability, *μ_eff_*, more accurately. Considering the series-connected inductive winding structure, Equation (4) can then be expressed as

(5) $$\Delta L=\frac{\mu_{0}\mu_{eff}\left(N_{1}^{2}+N_{2}^{2}\right)w_{mag}t_{mag}}{l_{mag}}.$$

Magnetic power loss is introduced by inserting a magnetic core inside the coils, which increases the total resistance, *R*, of the inductive windings in the HV-isolated transformer chips. Based on the classical theory of electromagnetism, the energy stored inside the core, *E_mag_*, and that of the magnetic power loss, *P_mag_*, are related to the real and imaginary parts of the permeability of the magnetic material used, respectively [29].

(6) $$E_{mag}=\frac{1}{2}∭\mu^{\prime }\left|H^{2}\right|dV$$

(7) $$P_{mag}=∭\omega \mu^{\prime \prime }\left|H^{2}\right|dV.$$

According to Lee’s assumption [30], if the real and imaginary parts of permeability are uniform inside the magnetic core, then *P_mag_* can be estimated as

(8) $$P_{mag}\approx 2\omega \left(\frac{\mu^{\prime \prime }}{\mu^{\prime }}\right)E_{mag},\;E_{mag}=\frac{1}{2}\Delta L\cdot I^{2}.$$

Based on Joule’s law, *P_mag_* can also be expressed as Equation (9), in which *R_mag_* represents the resistance contributed by magnetic power loss:

(9) $$P_{mag}=R_{mag}I^{2}.$$

Therefore, the total resistance, *R*, of the inductive windings in the HV-isolated transformer chip, which consists of *R_mag_* and air-core wingding resistance, *R_w_*, is given as

(10) $$R=R_{w}+R_{mag}=R_{w}+\omega \left(\frac{\mu^{\prime \prime }}{\mu^{\prime }}\right)\Delta L.$$

Based on the above equations, the inductance value *L_air_*, *L_mag_*, and total resistance (*R*) of the inductive windings are calculated as 105.9 nH, 770.6 nH, and 1.15 Ω, respectively, at a lower frequency of 3 MHz, for the designed device, with its geometry parameters listed in Table 1. In addition, when the operating frequency is well below its self-resonant frequency, the *Q*-factor can be expressed as *Q* = ω*L_mag_*/R [31] and is subsequently calculated as 12.6@3MHz.

To fully validate the device design and the above analytical calculations, finite element simulations were conducted by using COMSOL Multiphysics 5.5 software with the magnetic field module in the frequency domain. First, the structure of the HV-isolated transformer was modeled as a two-port network and driven by a 3 MHz excitation current, with an amplitude of 1 A through the one lumped port which connects the primary windings.

**Figure 2:**
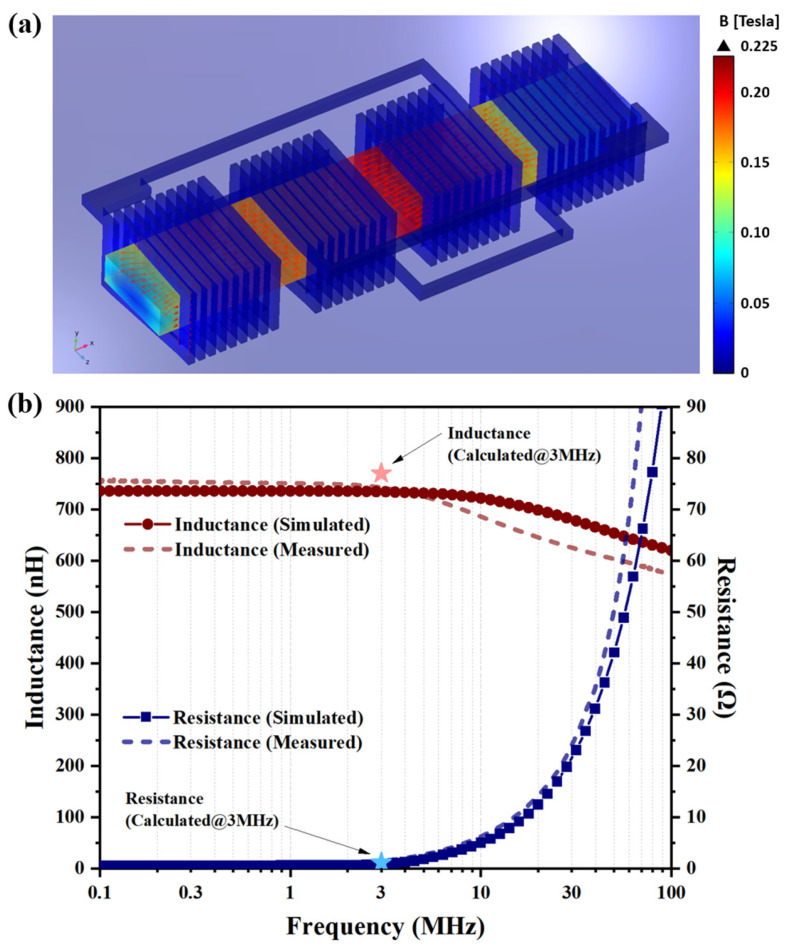
(**a**) Magnetic flux density colormap of the designed HV-isolated transformer chip under a 3 MHz excitation current, with an amplitude of 1 A loading on the primary windings. (**b**) Comparisons of inductance (*L*) and resistance (*R*) between calculations, finite element simulations, and subsequent measured results.

As shown in Figure 2a, when primary winding is excited, an intense magnetic field occurs inside the embedded magnetic core, with a maximum magnetic flux density of around 0.225 T, which is well below the saturation magnetic flux density of ferrite magnetic material, implying a higher current loading capability. Then, by applying excitations through two lumped ports sequentially, the two-port impedance matrix, which contains the inductance value and resistance of the transformer, is simulated over frequency. As plotted in Figure 2b, the finite element simulated inductance (*L*) and resistance (*R*) are consistent with the calculations from the design model, and they both show satisfactory agreement with the subsequent measured results in a wide frequency range, from 100 kHz to 100 MHz.

**Figure 3:**
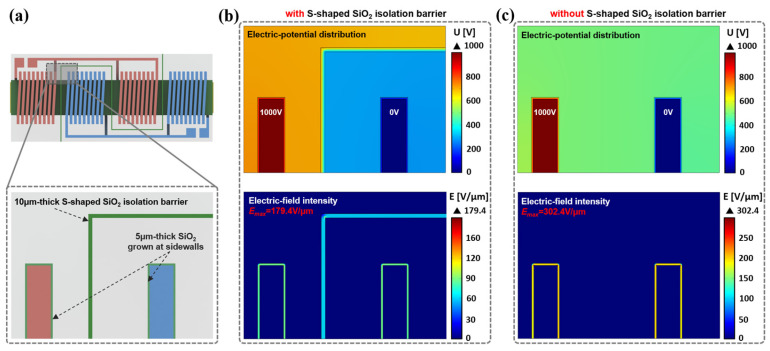
(**a**) Partially enlarged schematics demonstrating the SiO_2_ isolation barrier and the insulating layer grown at the sidewalls. (**b**) Finite element simulations of electric-potential distribution and electric-field intensity in HV-isolated transformer with embedded S-shaped isolation barrier and (**c**) the one without S-shaped isolation barrier under the application of 1000 V bias voltage.

The theoretical dielectric strength of typical insulator materials is compared in Table 2. SiO_2_ is preferred, as it provides an enormous dielectric strength of around 500 V/μm and superior mechanical stability. Shown in Figure 3a are the partially enlarged schematics demonstrating the designed structure of hybrid dielectric layers consisting of 10 μm thick S-shaped SiO_2_ isolation barriers and a 5 μm thick insulating layer grown at the silicon sidewalls.

The maximum electric-field intensity was then simulated to evaluate the theoretical breakdown voltage for the HV-isolated transformer under the bias voltage loading conditions. As shown in Figure 3b, when a 1000 V bias voltage is loaded on one side of an HV-isolated transformer with an integrated S-shaped isolation barrier, a well-controlled maximum electric-field intensity of 179.4 V/μm in the dielectric layer is found. As a comparison, under the same bias voltage loading condition, when the S-shaped isolation barrier is removed (as shown in Figure 3c), an increased maximum electric-field intensity of 302.4 V/μm is observed. Accordingly, based on the theoretical dielectric strength of SiO_2_, an ultra-high breakdown voltage of 2787.1 V is estimated for the HV-isolated transformer with the embedded S-shaped isolation barrier, which is about 70% higher than the 1653.4 V breakdown voltage for the transformer without an S-shaped isolation barrier.

## 3. Fabrication

**Figure 4:**
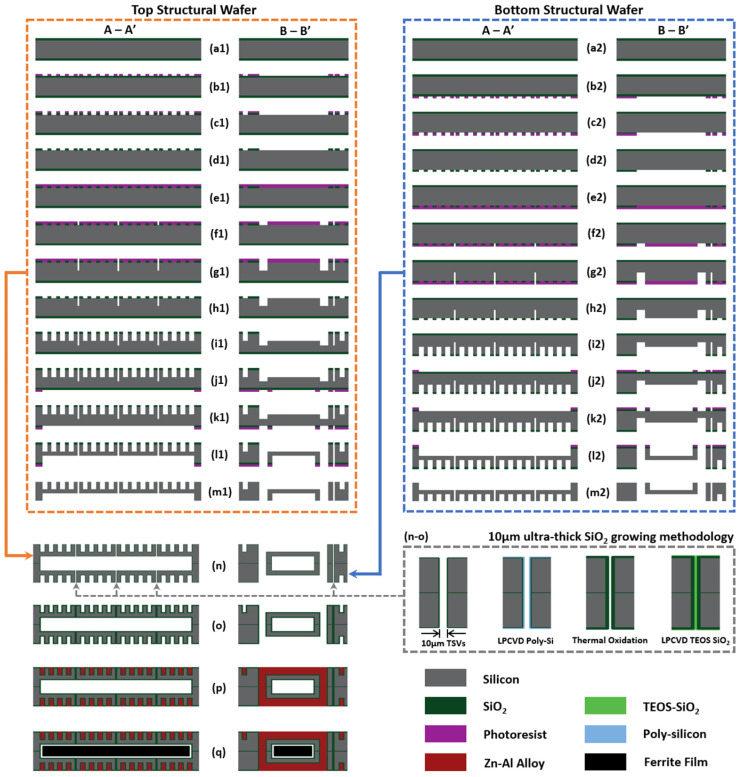
Fabrication processes flow for the HV-isolated transformers. (**a1**–**m1**) Top structural wafer and (**a2**–**m2**) bottom structural wafer formation by multiple photolithography and deep-RIE. (**n**–**o**) Silicon bonding and 10 μm thick SiO_2_ growing and filling inside the TSVs. (**p**) Molten alloy injecting via MEMS micro-casting process and (**q**) ferrite magnetic core inserting.

According to Figure 4 (the cutting planes A-A’ and B-B’ are indicated in Figure 1a,b), the fabrication process flow for the HV-isolated transformer chips contains two main stages, involving the structural wafers MEMS micromachining processes and the subsequent wafer-level MEMS micro-casting process for the metallization of three-dimensional high-density solenoidal structure within the aligned and bonded silicon structural wafers. Detailed fabrication steps are outlined as follows:

(a1–d1) For the preparation of the top structural wafer, a 1 μm thick SiO_2_ hard mask is first thermally grown on a 360 μm thick, 4-inch high-resistance (10,000 Ω·cm) silicon wafer and then patterned by using a front-side photolithography and SiO_2_ dry-etching process.

(e1–f1) The composite mask that defines the etching windows for both the S-shaped through-silicon vias measuring 10 μm in width and the vertical interconnection vias is formed after overlay photolithography on the patterned SiO_2_ layer.

(g1) The defined S-shaped vias, as well as the vertical interconnection vias, are then partially etched utilizing the deep reactive ion etching (deep-RIE) process.

(h1–i1) After the removal of the photoresist, deep-RIE is processed again to form the front-side horizontal grooves of the solenoidal cavities. Meanwhile, the S-shaped vias and the vertical interconnection vias are etched simultaneously.

(j1–k1) A double-side aligned photolithography and SiO_2_ dry-etching process is performed to pattern the backside of the SiO_2_ hard mask.

(l1–m1) With both patterned photoresist and SiO_2_ as etching masks, the cavity for magnetic core insertion is formed, and the vertical vias are etched through during the final deep-RIE process on the top structural wafer. After that, the photoresist and the SiO_2_ hard mask are removed.

(a2–m2) The bottom structural wafer is micromachined through the similar MEMS processes.

**Figure 5:**
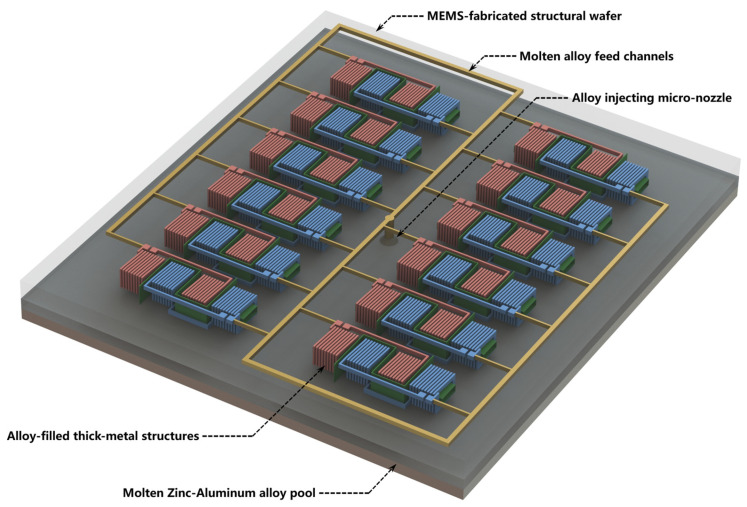
Partially enlarged schematics demonstrating the wafer-level batch formation for the three-dimensional thick-metal structures embedded inside the MEMS-fabricated 4-inch structural wafers during the developed Zn-Al molten alloy MEMS micro-casting process.

(n) After the etching for both the top and bottom structural wafers, 2 μm thick SiO_2_ layers are uniformly grown on the surface, as well as on the silicon sidewalls, of the structural wafers. Then, silicon-to-silicon direct hydrophilic bonding is carried out for the two aligned structural wafers and subsequently annealed in an oxygen atmosphere.

(o) Using the low-pressure chemical vapor deposition (LPCVD) process, 2 μm thick Poly-Si is uniformly deposited on both the surface and the sidewalls, followed by the application of a thermal oxidation process to fully convert the deposited Poly-Si into a thick SiO_2_ layer. Subsequently, 1 μm thick tetraethyl orthosilicate (TEOS) LPCVD SiO_2_ is deposited to finish filling the S-shaped vias.

(p) During the MEMS micro-casting process, micro-nozzles from above the liquid alloy pool are aligned with the inlet vias in the casting micro-mold. The molten Zn-Al alloy is then injected under exerting pressure through the micro-nozzles and guided by the alloy feed channels (see Figure 5) into the solenoidal cavities. As the exerting pressure slowly decreases under a specific rate to reach the rupture pressure of the liquid molten alloy bridge which is connecting the casting mold with the alloy pool, the alloy-filled thick-metal structures will then be pinched off from the micro-nozzles and then solidified inside the structural wafers under natural cooling.

**Figure 6:**
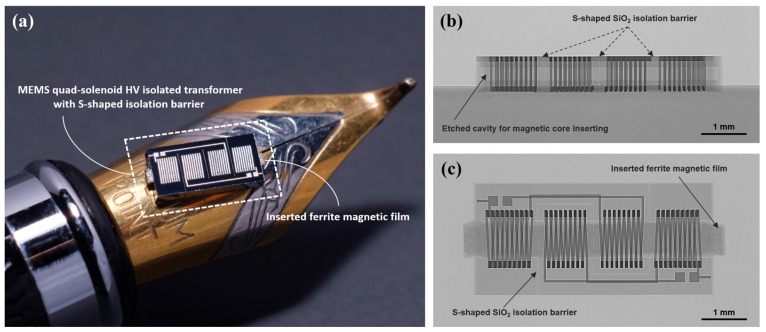
(**a**) Close-up view of the fabricated quad–solenoid HV-isolated transformer chip placed on a pen tip. (**b**) X-ray perspective micrograph showing the front view of the transformer chip. (**c**) X-ray perspective micrograph top view of the transformer chip with inserted magnetic core.

The fabricated quad–solenoid HV-isolated transformer chip is shown in Figure 6. Figure 6a is the macro-photograph of the transformer chip, which is saw-diced from a 4-inch wafer. The silicon-embedded solenoidal coupling coils occupy a tiny footprint of 6 mm^2^. Metal pads placed on each side of the chip are formed simultaneously during the micro-casting process, and they are designed for probe testing and the subsequent wire bonding.

Figure 6b is the X-ray perspective micrograph front view of the fabricated HV-isolated transformer chip, where a pre-etched cavity for magnetic core insertion centered in the silicon substrate can be clearly seen. From the X-ray perspective top view, which is shown in Figure 6c, a 200 μm thick ferrite magnetic core with a width of 700 μm and a length of 5100 μm is inserted into the pre-etched cavity. The S-shaped SiO_2_ isolation barriers completely separate the primary and secondary windings from the high-resistance silicon substrate.

## 4. Testing Results

**Figure 7:**
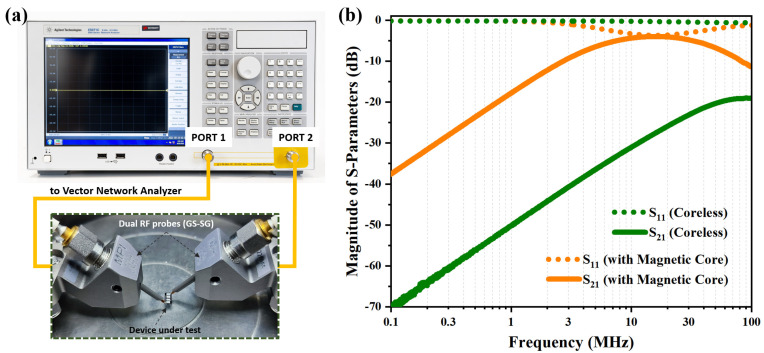
(**a**) Illustrations of the two-port scattering parameters (*S*-parameters) measurement setup for the fabricated HV-isolated transformers using a vector network analyzer (VNA) with a pair of RF probes. (**b**) The extracted two-port *S*-parameters of the HV-isolated transformers with an inserted ferrite magnetic core (orange line) and the one without a magnetic core (green line).

In order to further characterize the electromagnetic performance of the fabricated quad–solenoid HV-isolated transformer, including inductance integration density, coupling coefficient, and power transfer efficiency, two-port scattering parameters (*S*-parameters) of the chips were first measured and extracted. The measurement setup is shown in Figure 7a; the testing pads from primary and secondary windings are connected to the vector network analyzer through dual RF probes and coaxial cables. Prior to the measurement, standard SOLT calibrations, which include the short, open, load, and through calibration, are applied to effectively eliminate the effects of parasitic parameters. As plotted in Figure 7b, the extracted two-port *S*-parameters of the HV-isolated transformers with inserted ferrite magnetic core and the one without magnetic core were collected together.

**Figure 8:**
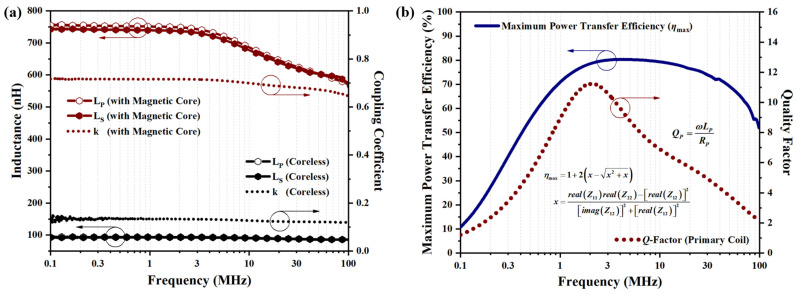
(**a**) Extracted frequency-dependent inductance values, as well as the coupling coefficient of the coupling coils in the fabricated HV-isolated transformer chips from the measured *S*-parameters. (**b**) Measured quality factor (*Q*-factor) and the achieved maximum power transfer efficiency versus frequency for the HV-isolated transformer with inserted ferrite magnetic core.

By converting the measured *S*-parameters to the impedance *Z*-matrix [37], the frequency-dependent inductance values, *L*, and the coupling coefficient *k* of the coupling coils in the transformer chips can then be extracted as follows:

(11) $$L_{P}=imag\left(Z_{11}\right)/\omega ,L_{S}=imag\left(Z_{22}\right)/\omega$$

(12) $$k=imag\left(Z_{12}\right)/\sqrt{imag\left(Z_{11}\right)\times imag\left(Z_{22}\right)}=L_{M}/\sqrt{L_{P}\times L_{S}}$$

where *L_P_*, *L_S_*, and *L_M_* represent the measured primary inductance, secondary inductance, and mutual inductance of the coupling coils, respectively. In Figure 8a, the extracted inductance values, as well as the coupling coefficient from the HV-isolated transformer with inserted magnetic core, are plotted and compared with the coreless one. Benefiting from the inserted magnetic core, enormous increments in *L_P_*, *L_S_*, and *k* are obtained, with the measurements improved from 93.3 nH, 92.1 nH, and 0.13 to 743.2 nH, 731.6 nH, and 0.72, respectively, at a targeted frequency of 3 MHz. Since the three-dimensional dense solenoidal coil structure occupies a tiny footprint of 6 mm^2^, its inductance integration density reaches 123.9 nH/mm^2^, which is well above the value that the previously reported monolithic transformer can offer [20,38,39,40,41]. In addition, attributed to the high depth-to-width ratio of the thick-metal coil structure formed by the MEMS micro-casting process, an extremely low DC resistance of only 0.39 Ω is measured by the four-probe method for the HV-isolated transformer. Thus, combined with the measured inductance value, an ultra-high inductance-to-resistance ratio (*L*/*R*) of over 1900 nH/Ω is achieved.

Plotted in Figure 8b are the measured *Q*-factor and the achieved maximum power transfer efficiency *η_max_* versus frequency for the HV-isolated transformer with inserted ferrite magnetic core. It can be seen that high *Q*-factor performance is obtained and reaches its peak of 11.2 at 2.1 MHz. At higher frequencies, the increase in AC resistance caused by the skin effect and eddy current losses in the magnetic core leads to a certain decrease in the *Q*-factor. The achieved peak *Q*-factor surpasses that of conventional single-layer planar monolithic transformers, which exhibit a *Q*-factor of only 7.8 [19]. As an essential figure of merit, the maximum power transfer efficiency is adopted to evaluate the efficiency of the HV-isolated transformer, and it is extracted from the impedance *Z*-matrix by Kiat T. Ng’s methods [42]. As shown in Figure 8b, a superior *η_max_* performance for the HV-isolated transformer of over 70% at a wide range of frequencies is achieved. At the targeted frequency of 3 MHz, the maximum power transfer efficiency even exceeds 80%, which implies a minimal capacitive power loss and a well-controlled eddy current loss in both the silicon substrate and in ferrite magnetic core.

**Figure 9:**
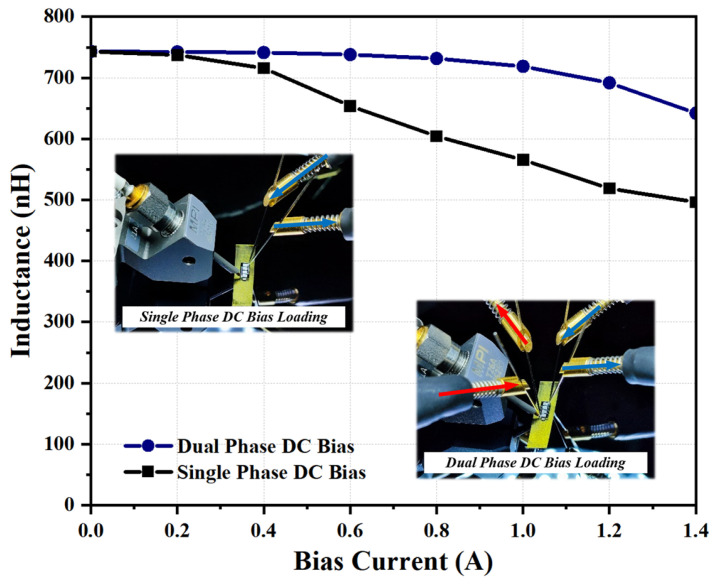
Measured inductance values of the HV-isolated transformer at 3 MHz with DC bias currents loading at primary and/or secondary windings. The insets demonstrate the probing setup.

**Figure 10:**
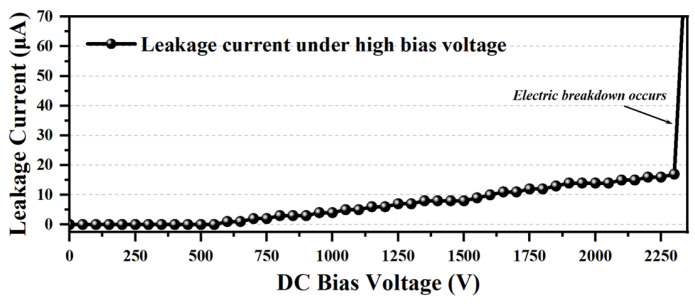
The measured leakage current as a function of high DC bias voltage applied between the primary and secondary windings in the HV-isolated transformer.

To assess the saturation current, *I_sat_*, of the transformer, its inductance values at 3 MHz as a function of DC bias current are measured and plotted in Figure 9. Under dual-phase DC bias currents loading condition, since the magnetic fluxes generated by the balanced dual phase bias currents will be largely canceled with each other [43], an ultra-high saturation current of over 1.4 A is measured when the inductance value of the transformer chip drops to 80% of its initial value. Meanwhile, under the single-phase DC bias current loading condition, the measured saturation current is 0.8 A, which is still double the saturation current reported in [7]. Furthermore, the insulation strength of the fabricated HV-isolated transformer chip is also measured through the breakdown voltage test, as the breakdown voltage of a transformer device indicates the maximum voltage it can withstand [20]. As shown in Figure 10, the transformer survived under fatally high DC bias voltage applied between the terminal from primary and secondary windings with a value of 2300 V during the breakdown voltage test. The measured withstand voltage is about 20% lower than the 2787.1 V predicted by the previous finite element simulation results, presumably caused by the fact that the actual dielectric strength of the SiO_2_ isolation barrier formed by thermal oxidation and oxidized LPCVD polysilicon is slightly lower than the material’s theoretical dielectric strength. Nevertheless, such a high withstand voltage of 2300 V is sufficient to cover most practical applications.

The detailed parameters of the proposed high-voltage-isolated MEMS quad–solenoid transformers are compared with those of previously reported monolithically integrated transformers in Table 3. Thanks to the novel structural design and the newly developed MEMS micro-casting technique, our devices not only achieve a higher inductance integration density and inductance to resistance ratio but can also provide better maximum power transfer efficiency in lower operating frequencies.

## 5. Conclusions

High-voltage-isolated MEMS quad–solenoid transformers with S-shaped SiO_2_ isolation barriers were designed, simulated, and wafer-level batch fabricated for compact isolated gate driver solutions in this study. Attributed to the silicon-embedded thick-metal dense coupling coil structures metalized through the MEMS micro-casting process, a high *Q*-factor of up to 11.2 and one of the highest inductance integration densities among the monolithically integrated transformers of 123.9 nH/mm^2^ were achieved. With the embedded 10 µm thick S-shaped SiO_2_ insulating barriers, the maximum electric-field intensity in the insulating dielectric layers from the transformer can be well controlled and, thus, allow for a higher withstand voltage of 2300 V, which was measured in the preliminary breakdown voltage test. Moreover, the developed transformers are characterized by superior power transfer efficiencies of over 80% and also exhibit a high saturation current under both single- or dual-phase DC bias loading conditions. Therefore, the superior electromagnetic performances combined with the achieved high saturation current and withstand voltage allow the devices to be well integrated into the miniaturized galvanically isolated gate driver solutions with highly improved power densities and efficiencies.

## Figures and Tables

**Table 1:** Summary of design parameters for HV-isolated quad–solenoid transformer chips.

Parameters	Primary Winding	Secondary Winding
Number of turns (*N*_1_ + *N*_2_)	18 (9 + 9)	18 (9 + 9)
Metal width	60 μm	60 μm
Metal depth	100 μm	100 μm
Gap between adjacent turns	40 μm	40 μm
Coil inner height	520 μm	520 μm
Coil inner width	900 μm	900 μm
**Parameters**	**Inserted thin-film ferrite magnetic core**	
Max relative permeability (at 3 MHz)	185 (real part, μ’); 13 (imaginary part, μ’’)	
Length of the magnetic core	5100 μm	
Width of the magnetic core	700 μm	
Thickness of the magnetic core	200 μm	

**Table 2:** Comparison of the dielectric strength of different types of insulator materials.

	Epoxy	Polyimide	BCB	LPCVD TEOS SiO_2_	Thermal SiO_2_
Dielectric strength	11.7 V/μm [32]	300 V/μm [33]	320 V/μm [34]	500 V/μm [35]	500 V/μm [36]

**Table 3:** Detailed comparisons between the proposed monolithically integrated HV-isolated transformers with previously reported related works.

Parameter	[38]	[39]	[40]	[41]	[20]	This Work
Magnetic core	Non	Yes	Yes	Non	Yes	Yes
Insulating materials	BCB	BCB	SiO_2_	SiO_2_	PI	SiO_2_
Frequency (MHz)	10	20	14	10	70	3
Inductance (nH)	159	80	114.9	113	47	743.2
Inductance density (nH/mm^2^)	79.5	22.85	8.64	56.5	10.44	123.9
Coupling coefficient	0.64	N/A	0.70	0.65	0.65	0.72
DC resistance (Ω)	0.4	0.34	0.15	0.31	0.5	0.39
*L/R* (nH/Ω)	398	235	766	360	94	1906
Isolation voltage (V)	4500	N/A	N/A	1050	500	2300
Max power transfer efficiency	70%	37%	N/A	70%	68%	80%

## Data Availability

The original contributions presented in the study are included in the article, further inquiries can be directed to the corresponding author.
